# EST and EST-SSR marker resources for *Iris*

**DOI:** 10.1186/1471-2229-9-72

**Published:** 2009-06-10

**Authors:** Shunxue Tang, Rebecca A Okashah, Marie-Michele Cordonnier-Pratt, Lee H Pratt, Virgil Ed Johnson, Christopher A Taylor, Michael L Arnold, Steven J Knapp

**Affiliations:** 1 Institute of Plant Breeding, Genetics, and Genomics, The University of Georgia, Athens, GA 30602, USA; 2 Laboratory for Genomics and Bioinformatics, The University of Georgia, Athens, GA 30602, USA; 3 Department of Genetics, The University of Georgia, Athens, GA 30602, USA

## Abstract

**Background:**

Limited DNA sequence and DNA marker resources have been developed for *Iris *(Iridaceae), a monocot genus of 200–300 species in the Asparagales, several of which are horticulturally important. We mined an *I. brevicaulis*-*I. fulva *EST database for simple sequence repeats (SSRs) and developed ortholog-specific EST-SSR markers for genetic mapping and other genotyping applications in *Iris*. Here, we describe the abundance and other characteristics of SSRs identified in the transcript assembly (EST database) and the cross-species utility and polymorphisms of *I. brevicaulis*-*I. fulva *EST-SSR markers among wild collected ecotypes and horticulturally important cultivars.

**Results:**

Collectively, 6,530 ESTs were produced from normalized leaf and root cDNA libraries of *I. brevicaulis *(IB72) and *I. fulva *(IF174), and assembled into 4,917 unigenes (1,066 contigs and 3,851 singletons). We identified 1,447 SSRs in 1,162 unigenes and developed 526 EST-SSR markers, each tracing a different unigene. Three-fourths of the EST-SSR markers (399/526) amplified alleles from IB72 and IF174 and 84% (335/399) were polymorphic between IB25 and IF174, the parents of *I. brevicaulis *× *I. fulva *mapping populations. Forty EST-SSR markers were screened for polymorphisms among 39 ecotypes or cultivars of seven species – 100% amplified alleles from wild collected ecotypes of Louisiana Iris (*I*.*brevicaulis*, *I*.*fulva*, *I. nelsonii*, and *I. hexagona*), whereas 42–52% amplified alleles from cultivars of three horticulturally important species (*I. pseudacorus*, *I. germanica*, and *I. sibirica*). Ecotypes and cultivars were genetically diverse – the number of alleles/locus ranged from two to 18 and mean heterozygosity was 0.76.

**Conclusion:**

Nearly 400 ortholog-specific EST-SSR markers were developed for comparative genetic mapping and other genotyping applications in *Iris*, were highly polymorphic among ecotypes and cultivars, and have broad utility for genotyping applications within the genus.

## Background

*Iris*, a genus of 200–300 species in the Iridaceae (Asparagales), is one of the most widely admired and earliest cultivated garden flowers, having appeared in ancient Eygptian artifacts as early as 1950 B.C. [1]. The most widely cultivated, hybridized, and horticulturally important species are *I*.*germanica *(tall-bearded Iris), *I*.*pseudacorus *(yellow-flag Iris), and *I*.*sibirica *(Siberian Iris), each with numerous commercially important cultivars. *Iris *species are found in diverse habitats on every continent in the Northern Hemisphere and have been important models for the study of plant evolution, ecology, and hybrid speciation [2-8]. Chromosome numbers and ploidy are highly variable among and within species in the genus, ranging from 2n = 16 in *I. attica *to 2n = 108 in *I. versicolor *[3,4]. Similarly, haploid genome lengths are generally large and highly variable in the genus, ranging from 2,000 to 30,000 Mbp [9].

Minimal genomic resources have been developed for *Iris*, a genus where forward genetic approaches have previously been applied to the study of life history and other traits by genotyping generic DNA markers, e.g., random amplified polymorphic DNA (RAPD) or retrotransposon display (IRRE) markers, in segregating populations developed from interspecific hybrids [7,9-14]. While such markers have facilitated linkage and quantitative trait locus (QTL) mapping in *Iris*, the uncertain orthology of RAPD and IRRE bands has precluded cross-referencing loci across populations and species. Simple sequence repeat (SSR), restriction fragment length polymorphism (RFLP), and single nucleotide polymorphism (SNP) markers are typically ortholog-specific and, consequently, have been widely used as DNA landmarks for synteny analysis and cross-referencing loci across populations [15-17]. Thus far, a limited number of ortholog-specific DNA marker have been described for *Iris *[18]. The primary goal of the present study was to develop a sufficient number of ortholog-specific DNA markers for genome-wide comparative genetic mapping and other genotyping applications in *I. brevicaulis *(x = 20), *I. fulva *(x = 20), and other species in the genus by developing a small EST database and targeting SSRs in ESTs.

SSRs are ubiquitous in transcribed sequences, typically locus-specific and co-dominant, and often multi-allelic, highly polymorphic, and transferrable among species within genera [19-22]. EST databases have been a rich source of SSRs for the development of ortholog-specific EST-SSR markers for genotyping applications in numerous species of flowering plants [21-28]. When our study was initiated, a limited number of ESTs (201) had been deposited in GenBank  for a single species in the genus, *I. hollandica *[29], and were insufficient for EST-SSR marker development. We developed a small EST database from cDNA sequences produced from normalized cDNA libraries of two species of Louisiana Iris (*I. brevicaulis *and *I. fulva*), partly to support the development of several hundred EST-SSR markers for comparative mapping and other genotyping applications in Louisiana Irises and partly to create DNA sequence and ortholog-specific DNA markers resources for the genus as a whole. Previous forward genetic analyses in *I. brevicaulis *and *I. fulva *identified QTL for several morphological, life history, and ecological traits [12,13,30-32]. Because ortholog-specific DNA markers were previously lacking for these genera, linkage groups and QTL identified in earlier analyses could not be cross-referenced and comparative genetic mapping was infeasible. Here, we describe the *I. brevicaulis*-*I. fulva *EST database and the development, cross-species utility, and polymorphisms of *I. brevicaulis*-*I. fulva *EST-SSR markers among wild collected ecotypes of four species of Louisiana Iris (*I. brevicaulis*, *I. fulva*, *I. hexagona*, and *I. nelsonii*) and horticulturally important cultivars of tall-bearded (*I*.*germanica*), yellow-flag (*I*.*pseudacorus*), and Siberian (*I*.*sibirica*) Iris.

## Results and Discussion

### Development of a Leaf and Root EST Database for *Iris*

Normalized leaf and root cDNA libraries were developed from *I*.*brevicaulis *(IB72) and *I*.*fulva *(IF174) ecotypes (root and leaf RNAs were pooled and a single cDNA library was constructed for each species). Library quality was checked by sequencing colony-PCR amplified inserts from 295 randomly selected cDNA clones split between the IB72 and IF174 libraries. Of the 295 clones, 251 (85.1%) harbored inserts 800 bp or longer, three lacked inserts (1.1%), and 290 (98.3%) harbored unique inserts. Subsequently, 12,199 cDNA clones were single-passed sequenced and yielded 6,530 ESTs surpassing quality standards, 2,947 from the IB72 and 3,583 from the IF174 library. Less than 1% of the clones lacked cDNA inserts (85/12,199). The vector- and quality-trimmed ESTs were deposited in GenBank (Acc. No. EX949962–EX956238 and FD387191–FD387443), annotated by BLASTX analyses against NCBI databases, assembled, and deposited in a database  developed by modifying a previously described EST processing pipeline and database [33]. The mean length of vector- and quality-trimmed ESTs was 578.0 bp.

cDNA normalization minimized redundancy in the *Iris *EST database and yielded a wealth of unique cDNA sequences (unigenes) for EST-SSR marker development and other applications in *Iris *biology, breeding, and floriculture . The 6,530 ESTs assembled into 4,917 unigenes (3,851 singletons and 1,066 contigs); hence, 75.3% of the ESTs were unique and 78.3% of the unigenes were singletons. cDNAs were normalized using a protocol which has been applied in numerous plant and animal species and minimized abundant transcript resequencing [34-36]. cDNA populations in leaves are dominated by abundant transcripts, e.g., chlorophyl A/B binding proteins and rubisco, neither of which were abundant among transcripts isolated by sequencing normalized leaf cDNA libraries. The deepest contigs contained seven ESTs.

Unigenes ranged in length from 100 to 1,673 bp with a mean length of 603.1 bp. Less than one-tenth of the unigenes (433/4,917) were sequenced through the polyA tail. Mean GC contents, which were identical for *I*.*brevicaulis *(45.4%) and *I*.*fulva *(45.3%), were slightly greater than mean GC contents reported for onion (*Allium cepa *L.; 41.9%) and *Arabidopsis *(42.7%) transcripts [37,38]. Unigenes were annotated by BLASTX  analyses against the NCBI Non-Redundant Protein  and UniProtKB Swiss-Prot and TrEMBL  databases. Using a BLASTX threshold of <*E *= 1^10^, significant similarities were found and putative functions were identified for 2,390 *Iris *unigenes (48.6%). Thirty-two (0.6%) additional unigenes were similar to genes of unknown function. Significant similarities were not found for the other 2,495 *Iris *unigenes (50.8%). The fraction of unigenes homologous to cDNAs encoding known function genes was similar to onion, an economically important species in the Asparagales [38].

The Louisiana Iris ESTs developed in the present study have moderately increased DNA sequence resources for *Iris*, which were previously minimal , and supplied ESTs for an important basal species in the Asparagales, a family where DNA sequence information has primarily been produced for onion, asparagus (*Asparagus officinalis *L.), and model species [38,39]. van Doorn et al. [29] previously described 201 *I. hollandica *ESTs from a tepal cDNA library. Other than the latter, 607 nucleotide sequences for 104 species of *Iris *had previously been deposited in public databases, the bulk of which were for a limited number of DNA sequence motifs commonly targeted in phylogenetic analyses, e.g., matK. The Sanger ESTs described here were produced before the emergence of next-generation DNA sequencing technologies, which have dramatically increased DNA sequencing throughput and are facilitating deeper and broader DNA sequencing than was previously practical in species with limited DNA sequence resources [40-42]. The Sanger ESTs we produced, while limited in number, build the foundation for deeper transcriptome sequencing in *Iris *using next-generation technologies.

### Abundance, Characteristics, and Distribution of SSRs in Louisiana Iris ESTs

**Figure 1:**
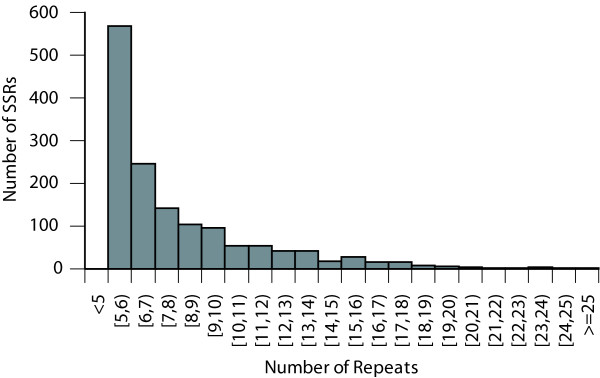
**Distribution of repeat counts for simple sequence repeats (SSRs) identified in 1,162 unigenes in the *I. brevicaulis-I. fulva *EST database**.

**Figure 2:**
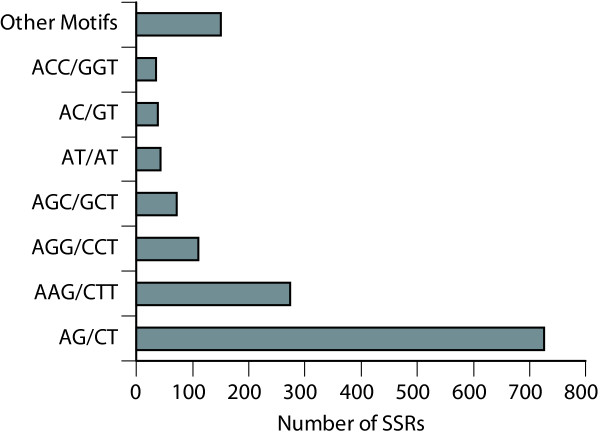
**Distribution of dinucleotide and trinucleotide repeats identified in 1,162 unigenes in the *I. brevicaulis-I. fulva *EST database**.

SSRs were highly frequent in the Louisiana Iris EST database (Figures 1, 2; Additional File 1; ). We identified 1,447 perfect SSRs (*n *≥ 5) in 1,162 unigenes. One-fourth of the 4,917 unigenes in the transcript assembly harbored at least one SSR, a frequency which was much greater than the frequency range (2–12%) in many other flowering plants [20,21,24,43]. The mean SSR density was one per 2,048 bp, which was much higher than the density found in onion (1/25 kb; [38]), another species in the Asparagales, and *Arabidopsis *(1/14 kb; [44]). When the transcript assembly was mined for perfect and imperfect repeats, 3,487 SSRs (*n *≥ 5) were identified in 2,037 unigenes (41.4%) with a mean density of approximately one SSR per 850 bp; imperfect repeats are interrupted short tandem repeats.

SSR repeat numbers ranged from 5 to 30 and lengths ranged from 10 to 69 bp (Figure 1; Additional File 1). Of the 1,447 perfect SSRs, 1,077 (72.9%) were 14 bp or longer and 694 (48.0%) were 18 bp or longer. The mean repeat number was 9 and the mean repeat length was 23 bp. Of the 1,447 perfect repeats, 807 were dinucleotides (55.8%) and 569 were trinucleotides (39.3%). The most common repeat motifs were AG/CT (50.1%), AAG/CTT (18.9%), and AGG/CCT (7.6%) (Figure 2). Slightly more than two-thirds of the SSRs were located in UTRs (61.4% in 5'-UTRs, 8.3% in 3'-UTRs, and 30.3% in exons). Of repeats identified in UTRs, 62.3% were dinucleotides and 31.4% were trinucleotides. Conversely, of repeats identified in CDSs, 17.4% were dinucleotides and 82.6% were trinucleotides (Additional File 1). The low frequency of SSRs identified in 3'-UTRs was primarily a function of 5'-end sequencing, which yielded significantly fewer 3' than 5'-UTR sequences (8.8% of the unigenes harbored polyA tails).

The most common dinucleotide repeat motif was AG/CT, which constituted 89.8% of the dinucleotide repeats identifed in *Iris *ESTs and has been the most common dinucleotide repeat identified in other plant EST databases [20,21,24,43]. AG/CT repeats have been widely targeted for EST-SSR marker development in plants because, in addition to being highly abundant, they are often highly polymorphic, more abundant in UTRs than CDSs, seldom associated with transposons, and consistently amplify and yield robust SSR markers [20,24]. The frequencies of trinucleotide repeats in CDSs and dinucleotide repeats in UTRs appear to be similar in wheat and *Iris *([26]; Additional File 1).

Trinucleotide repeats are typically more abundant than dinucleotide repeats in plants [21]; however, dinucleotide repeats (56%) were more abundant than trinucleotide repeats (39%) in *Iris*. Trinucleotide repeats (54–78%) have been more abundant than dinucleotide repeats (17–40%) in analyses of EST databases of several grass species [22,27,43,45]. Of the EST-SSRs identified in wheat (*Triticum aestivuum *L.), 70% were trinucleotides and 30% were dinucleotides [27]. AAG/CTT and AGG/CCT (67.5%) were the most abundant trinucleotide repeats in *Iris*, whereas GCC/GGC appears to be the most abundant trinucleotide repeat motif in other plants [19,20,24,45]. SSRs were more abundant in UTRs than CDSs in *Iris*, whereas they are more abundant in CDSs than UTRs in other plant species [21,24,43,46]. SSR abundance in 3'-UTRs of *Iris *ESTs may have been underestimated in the present study by 5' directional sequencing of cDNAs, which artificially skews the distribution. SSRs appear to be equally abudant in 5'- and 3'-UTRs in other plant species [20,25]. If this pattern holds in *Iris*, the frequency of SSRs in UTRs could be as great as 80%, which implies the frequency of SSRs in *Iris *ESTs could be greater than reported here (Additional File 1).

### Louisiana Iris EST-SSR Marker Development, Screening, Allele Length Polymorphisms, and Cross-Species Utility

SSRs with *n *≥ 6 repeats were selected from 526 unigenes for primer design and marker development (SSR primer sequences, allele lengths, repeat motifs, and other characteristics of the SSR markers are supplied in Additional File 2). The SSR markers were initially screened for amplication and allele length polymorphisms among three Lousiana Iris ecotypes (IB25, IB72, and IF174). Of the 526 EST-SSR markers, 399 (76%) amplified alleles from at least one genotype (the null allele frequency was 2.7%), whereas 127 (24%) either failed to amplify alleles or produced amplicons which were too long (> 700 bp) or complex for genotyping. Of the 399 EST-SSR markers, 72 spanned introns longer than 200 bp and amplified alleles longer than 700 bp and could not be genotyped (Additional File 2). Of the 327 SSR markers found to amplify alleles within the prescribed genotyping length range (100–700 bp), 283 (87%) were polymorphic among IB25, IB72, and IF74. The number of polymorphic SSR markers/cross ranged from 247 (76%) for IB25 × IB72 to 276 (84%) for IB72 × IF174 (Additional File 2). Hence, most of the EST-SSR markers were polymorphic in *I*.*brevicaulis *× *I*.*fulva *mapping populations.

**Figure 3:**
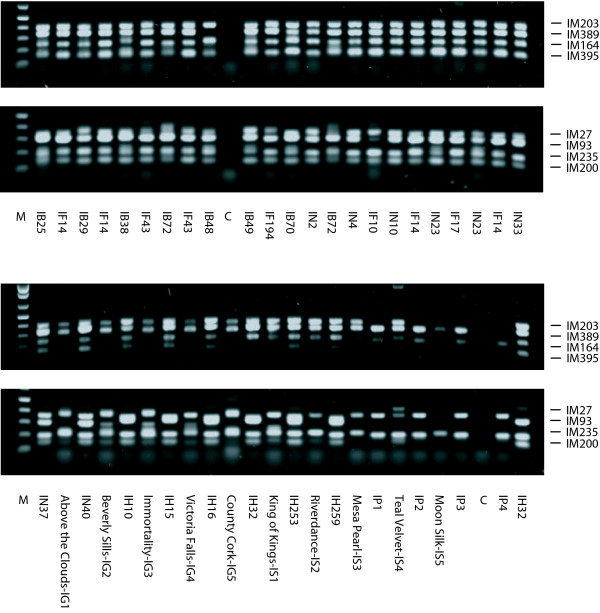
**Genotypes for two multiplexes of four EST-SSR markers each screened for amplification and length polymorphisms on agarose among 39 ecotypes or cultivars of *I. brevicaulis *(IB), *I. fulva *(IF), *I. nelsonii *(IN), *I. hexagona *(IH), *I. pseudacorus *(IP), *I. germanica *(IG), and *I. sibirica *(IS)**. EST-SSR markers in the multiplexes were IM203, IM389, IM164, and IM395 or IM27, IM93, IM235, and IM200.

Forty *I*.*brevicaulis*-*I*.*fulva *EST-SSR markers were selected for more in-depth screening and analysis, primarily to quantify polymorphisms and assess transferability and utility among a broader sample of ecotypes, cultivars, and species. The 40 EST-SSR markers, which have been genetically mapped in *I*.*brevicaulis *× *I*.*fulva *and are distributed across the genome (unpublished data), were screened for amplification and allele length polymorphisms among 26 wild collected ecotypes of four Lousiana *Iris *species (*I. brevicaulis*, *I. fulva*, *I. hexagona*, and *I. nelsonii*) and 13 cultivars of tall-bearded (*I*.*germanica*), yellow-flag (*I*.*pseudacorus*), and Siberian (*I*.*sibirica*) Iris (Tables 1, 2; Additional File 3). Whilst 100% of the EST-SSR markers amplified alleles from Louisiana Iris ecotypes, half or slightly less than half amplified alleles from yellow-flag (52.5%), Siberian (45.0%), and tall-bearded (42.5%) Iris cultivars. Of the 40 EST-SSR markers, only nine amplified alleles across the 39 ecotypes and cultivars (Figure 3; Additional File 4). One to three alleles/marker were amplified from triploid *I*.*germanica*, whereas one to two alleles/marker were amplified from the diploid species (Additional File 4). The nine EST-SSR markers were highly polymorphic among tall-bearded, yellow-flag, and Siberian Iris cultivars; heterozygosities ranged from 0.77 to 0.91 (Table 2). Even though *I. pseudacorus *and *I. sibirica *belong to the same section (Limniri) as Louisiana Iris [5], a significant decrease in allele amplification was observed in these species, and was comparable to the decrease observed in *I. germanica*, a species from section Iris. Nevertheless, many of the *I*.*brevicaulis*-*I*.*fulva *EST-SSR markers developed in the present study amplify alleles from other species and should have broad utility in the genus [1,5].

**Table 1:** Linkage group (LG) assignment, number of alleles (*n*), and mean heterozygosity (*h*) estimated from genotypes of 40 EST-SSR markers among seven *I. brevicaulis *(*n*_*B *_and *h*_*B*_), six *I. fulva *(*n*_*F *_and *h*_*F*_), six *I. hexagona *(*n*_*H *_and *h*_*H*_), and seven *I. nelsonii *(*n*_*N *_and *h*_*N*_) ecotypes and among 26 Louisiana Iris ecotypes (*n*_*L *_and *h*_*L*_).

**SSR** **Marker**	**Motif**	**SSR Location**	**LG**	***n*_*B*_**	***n*_*F*_**	***n*_*H*_**	***n*_*N*_**	***n*_*L*_**	***h*_*B*_**	***h*_*F*_**	***h*_*H*_**	***h*_*N*_**	***h*_*L*_**
IM27	(AAG)12	5' UTR	18	2	1	1	1	4	0.24	0.00	0.00	0.00	0.64
IM55	(GAA)9	CDS	1	6	7	4	1	10	0.76	0.83	0.68	0.00	0.82
IM61	(AGC)7	CDS	4	5	3	2	3	8	0.71	0.40	0.49	0.36	0.69
IM93	(ACC)8	CDS	9	6	2	2	2	6	0.76	0.38	0.28	0.13	0.72
IM95	(AAG)8	5' UTR	17	6	1	3	3	11	0.80	0.00	0.50	0.65	0.82
IM123	(TCT)10	CDS	3	7	4	2	5	8	0.83	0.65	0.50	0.61	0.81
IM144	(CAG)6	CDS	3	5	1	2	2	5	0.67	0.00	0.49	0.41	0.64
IM146	(ACG)6	CDS	5	4	2	2	4	6	0.72	0.15	0.38	0.53	0.77
IM152	(CTA)6	CDS	6	4	3	2	2	5	0.37	0.57	0.28	0.34	0.64
IM156	(GAG)7	CDS	4	4	3	2	1	6	0.62	0.29	0.15	0.00	0.36
IM164	(AGC)6	CDS	12	2	2	3	4	7	0.46	0.44	0.57	0.66	0.82
IM165	(CAG)6	3' UTR	5	3	1	3	2	5	0.64	0.00	0.57	0.24	0.72
IM196	(AAG)7	CDS	10	6	5	3	7	16	0.68	0.72	0.54	0.83	0.90
IM200	(GAA)23	CDS	1	9	4	2	3	10	0.87	0.51	0.28	0.44	0.77
IM203	(CTT)7	5' UTR	13	7	2	3	3	9	0.80	0.50	0.40	0.60	0.75
IM234	(GCA)7	CDS	2	3	1	4	5	8	0.50	0.00	0.71	0.67	0.70
IM235	(TGC)6	3' UTR	12	4	2	4	3	10	0.66	0.44	0.58	0.62	0.83
IM283	(CCT)8	CDS	21	2	2	2	1	4	0.13	0.15	0.15	0.00	0.55
IM299	(TA)6	3' UTR	19	4	4	3	4	7	0.66	0.51	0.57	0.73	0.80
IM303	(TCC)6	5' UTR	16	6	5	2	1	10	0.80	0.69	0.15	0.00	0.77
IM319	(GAG)7	5' UTR	5	9	1	3	3	11	0.87	0.00	0.29	0.52	0.82
IM327	(GA)12	5' UTR	20	6	7	4	4	13	0.78	0.79	0.60	0.70	0.88
IM341	(GAG)6	5' UTR	12	3	1	3	1	7	0.50	0.00	0.65	0.00	0.69
IM348	(AGG)7	CDS	2	7	3	8	4	11	0.70	0.49	0.85	0.46	0.77
IM364	(CTT)6	CDS	9	9	4	2	2	10	0.87	0.65	0.15	0.24	0.73
IM377	(ATT)6	5' UTR	7	3	2	1	2	7	0.26	0.44	0.00	0.41	0.78
IM378	(TTC)9	5' UTR	6	3	3	2	2	6	0.50	0.57	0.15	0.50	0.78
IM389	(CTT)8	CDS	14	8	3	1	4	9	0.84	0.29	0.00	0.66	0.70
IM391	(TTC)12	5' UTR	15	6	7	2	5	12	0.69	0.83	0.15	0.73	0.87
IM395	(TC)8	3' UTR	15	3	3	3	4	7	0.57	0.61	0.57	0.53	0.80
IM402	(CTT)10	5' UTR	13	7	2	2	3	9	0.82	0.49	0.15	0.60	0.78
IM426	(TCT)7	5' UTR	4	6	4	2	4	11	0.76	0.71	0.38	0.62	0.85
IM429	(AGA)8	5' UTR	14	3	3	3	2	8	0.44	0.40	0.40	0.34	0.75
IM442	(GA)7	5' UTR	16	2	1	1	2	2	0.34	0.00	0.00	0.46	0.47
IM450	(CAG)7	CDS	2	7	2	5	4	10	0.74	0.28	0.53	0.64	0.70
IM460	(GA)7	5' UTR	8	7	2	3	4	13	0.83	0.28	0.54	0.66	0.85
IM486	(AGC)7	CDS	11	3	5	4	7	12	0.57	0.72	0.68	0.80	0.89
IM501	(AG)13	5' UTR	7	6	3	5	4	14	0.76	0.40	0.72	0.61	0.88
IM503	(CT)12	5' UTR	10	7	6	5	7	18	0.82	0.82	0.68	0.76	0.89
IM526	(CAC)6	CDS	11	6	3	5	3	9	0.73	0.40	0.76	0.52	0.83
Mean				5.2	3.0	2.9	3.2	8.9	0.65	0.41	0.41	0.47	0.76

**Table 2:** Number of alleles (*n*) and heterozygosities (*h*) estimated from genotypes of nine EST-SSR markers among 26 Louisiana Iris ecotypes (*n*_*L *_and *h*_*L*_), 13 yellow-flag, Siberian, and tall-bearded Iris cultivars (*n*_*C *_and *h*_*C*_), and across the 39 ecotypes and cultivars (*n*_*T *_and *h*_*T*_).

**SSR** **Marker**	**Motif**	**SSR Location**	**LG**	***n*_*L*_**	***n*_*C*_**	***n*_*T*_**	***h*_*L*_**	***h*_*C*_**	***h*_*T*_**
IM61	(AGC)7	CDS	4	8	10	15	0.69	0.82	0.88
IM93	(ACC)8	CDS	9	6	6	8	0.72	0.77	0.79
IM123	(TCT)10	CDS	3	8	6	14	0.81	0.82	0.91
IM164	(AGC)6	CDS	12	7	7	13	0.82	0.79	0.88
IM196	(AAG)7	CDS	10	16	10	20	0.90	0.87	0.93
IM200	(GAA)23	CDS	1	10	8	18	0.77	0.83	0.90
IM327	(GA)12	5' UTR	20	13	15	27	0.88	0.91	0.94
IM348	(AGG)7	CDS	2	11	8	15	0.77	0.86	0.88
IM391	(TTC)12	5' UTR	15	12	9	17	0.87	0.87	0.93
Mean				10.1	8.8	16.3	0.80	0.84	0.89

The 40 EST-SSR markers were highly polymorphic among and consistently amplified alleles from Louisiana Iris ecotypes; the null allele frequency was 0.5% (Table 1; Figure 3; Additional File 4). The number of alleles/locus (*n*) ranged from two to 18, the mean number of alleles/locus (*n*) was 8.9, heterozygosities of individual SSR markers ranged from 0.36 to 0.90, and the mean heterozygosity (*h*) was 0.76. Eighty to 100% of the EST-SSR markers were polymorphic, *n *ranged from 2.9 to 5.2, and *h *ranged from 0.41 to 0.65 among Louisiana Iris ecotypes (Table 1; Additional File 4). The number of species-specific alleles ranged from 26 in *I. nelsonii *to 101 in *I. brevicaulis*. Dinucleotide repeats were slightly more polymorphic than trinucleotide repeats. The mean number of alleles was *n *= 10.6 for dinucleotide and 8.5 for trinucleotide repeats and the mean heterozygosity was *h *= 0.80 for dinucleotide and 0.75 for trinucleotide repeats. SSRs in coding sequences (*h *= 0.78) were only slightly less polymorphic than SSRs in UTRs (*h *= 0.73) (Table 1; Additional File 1).

### Genetic Diversity Among Wild Collected Ecotypes and Horticulturally Important Cultivars

**Figure 4:**
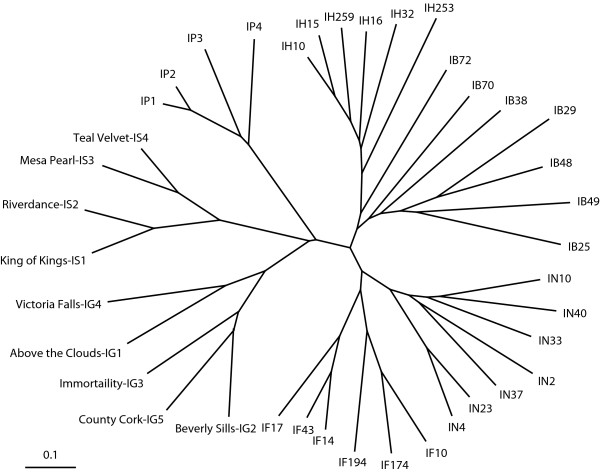
**Dendrogram constructed from genetic distances estimated from genotypes of nine EST-SSR markers among seven *I. brevicaulis *(IB), six *I. fulva *(IF), six *I. hexagona *(IH), and seven *I. nelsonii *(IN) ecotypes and four *I. pseudacorus *(IP), five *I. germanica *(IG), and four *I. sibirica *(IS) cultivars**.

Because only nine of the 40 *I. brevicaulis*-*I*.*fulva *EST-SSR markers amplified alleles from horticultural cultivars of *I*.*germanica*, *I*.*pseudacorus*, and *I*.*sibirica*, genetic distances and dendrograms were separately estimated from genotypes of the nine EST-SSR markers among accessions of all seven species and of the 40 EST-SSR markers among ecotypes of the four Louisiana Iris species (Figure 4; Additional File 4). Genetic distances (*G*) ranged from 0.25 to 0.93 among Louisiana Iris ecotypes. The longest genetic distances were interspecific (*G *= 0.93 between IB70 and IF10, IH32 and IN33, and IB25 and IF17), whilst the shortest genetic distances were intraspecific (*G *= 0.25 between IF14 and IF17 and IH10 and IH16). Ecotypes assembled into species-specific clusters which were separated by greater genetic distances than ecotypes within species-specific clusters (Figure 4; Additional File 5). Genetic diversity was significant and diffuse among ecotypes or cultivars within species. Only a few EST-SSR markers were needed to identify (distinguish) ecotypes and cultivars.

## Conclusion

cDNA sequences, an EST database, and EST-SSR markers were developed for comparative mapping, forward genetics, and other genotyping applications in *Iris*. cDNA normalization minimized transcript redundancy and small scale EST sequencing (6,530) yielded 4,917 unigenes for gene discovery and DNA marker development. Perfect SSRs were identified in one-fourth of the unigenes (1,162/4,917) and EST-SSR markers were developed for nearly half of the latter (526/1,162). Three-fourths of the primers designed and tested (399/526) amplified alleles from reference ecotypes of *I. brevicaulis *and *I. fulva *and yielded robust EST-SSR markers. When 40 of the EST-SSR markers were screened for amplification, genotyping utility, and polymorphisms across species, 100% amplified alleles from *I. brevicaulis*, *I. fulva*, *I. hexagona*, and *I. nelsonii *ecotypes, whereas 42–50% amplified alleles from *I*.*germanica*, *I*.*pseudacorus*, and *I*.*sibirica *ecotypes and cultivars. Hence, a large percentage of the *I. brevicaulis*-*I. fulva *EST-SSR markers developed in the present study should amplify alleles from other species and have broad utility in the genus. Finally, significant allelic diversity was discovered among Louisiana, yellow-flag, Siberian, and tall-bearded ecotypes and cultivars; 90% of the EST-SSR markers were polymorphic and supply a wealth of ortholog-specific DNA markers for biological and horticultural research in *Iris*.

## Methods

### cDNA Library Construction

Normalized cDNA libraries were constructed from RNAs isolated from leaves and roots of an *I*.*brevicaulis *ecotype (IB72) and an *I*.*fulva *ecotype (IF174) using the Creator SMART cDNA Library Construction Kit (Clontech, Mountain View, CA). Total RNAs were isolated using Trizol (Invitrogen, Carlsbad, CA). First-strand cDNAs were synthesized using SuperScript™ III Reverse Transcriptase (Invitrogen, Carlsbad, CA). Double-strand cDNAs were normalized by duplex-specific nuclease (DSN) purified from Kamchatka crab hepatopancreas using the Trimmer-Direct cDNA Normalization Kit (Evrogen, Moscow, Russia). Normalized cDNAs were size-fractionated, and cDNAs > 400 bp were selected for cDNA library construction. Normalized and size selected cDNAs were digested with *Sfi *I (Fermentas, Glen Burnie, MD), directionally cloned into the vector pDNR-LIB, and electroporated into competent cell DH10B (Clontech, Mountain View, CA). To assess cDNA library quality and insert length distribution, inserts were amplified from 295 randomly selected cDNA clones by colony PCR.

### EST Database Development

Collectively, 12,199 *I. brevicaulis *and *I. fulva *cDNA clones were 5'-end single-pass Sanger sequenced at the Washington University Genome Sequencing Center, St. Louis, MO using M13 as the sequencing primer; roughly equal numbers of randomly selected clones were sequenced from the two cDNA libraries. ESTs were processed, vector- and quality-trimmed, assembled, and annotated using a custom bioinformatics pipeline and deposited and displayed in *Iris *ESTdb , a relational EST database developed by modifying a previously described database [33]. Low-quality bases were PHRED-trimmed  using a Q < 16 quality score (Q) threshold. Vector sequences were trimmed using Cross_Match . cDNA sequences were screened for *E. coli*, chloroplast, and mitochondrial DNAs utilizing the SSAHA package . Vector- and quality-trimmed ESTs longer than 100 bp were assembled using MEGABLAST and CAP3 TGI Clustering Tools . BLASTX  analyses were performed against the NCBI Non-Redundant Protein Database, UniprotSprot, and UniprotTrembl to identify putative functions of and annotate unique transcripts (unigenes).

### EST-SSR Discovery, Marker Development, and Length Polymorphism Screening

Unigenes in the transcript assembly were screened for perfect repeat motifs using SSR-IT (; [24]) and imperfect repeat motifs using FastPCR . SSRs with a minimum repeat count (*n*) threshold of *n *≥ 5 were selected for further analysis and EST-SSR marker development (Additional File 1). Flanking forward and reverse primers were designed for SSRs in 526 unigenes using Primer 3 (; Additional File 2). To facilitate multiplex genotyping on an ABI3730 XL Capillary DNA Sequencer (Applied Biosystems, Foster City, CA), SSR primers were designed by uniformly varying target amplicon lengths from 100 to 450 bp and end-labeling forward primers with one of three fluorophores, 6FAM, HEX, or TAMRA (Additional File 2). The 526 SSR markers were screened for amplification and length polymorphisms among two *I*.*brevicaulis *ecotypes (IB72 and IB25) and an *I*.*fulva *ecotype (IF174) on agarose [47].

To assess cross-species amplification, transferability, and allele length polymorphisms, 40 of the 526 *I. brevicaulis-I. fulva *EST-SSR markers were screened among 26 ecotypes sampled from four Louisiana Iris species (*I*.*brevicaulis*, *I*.*fulva*, *I. nelsonii*, and *I. hexagona*), four yellow-flag Iris cultivars (*I*.*pseudacorus*), four Siberian Iris cultivars (*I*.*sibirica*), and five tall-bearded Iris cultivars (*I*.*germanica*) (Additional File 3). Louisiana Iris ecotypes were collected from Terrebonne Parish and St. Martinville Parish, Louisiana and *I*.*pseudacorus *ecotypes were collected from Spring Lake, San Marcos, TX. Tall-bearded and Siberian Iris cultivars were purchased from Schreiner Iris Gardens, Salem, Oregon. The 40 EST-SSR markers were previously mapped and distributed among 21 *I*.*brevicaulis *× *I*.*fulva *linkage groups (unpublished data). Genomic DNA was isolated from leaves of the 39 ecotypes or cultivars using a modified cetyltrimethylammonium bromide (CTAB) method [48]. SSR markers were genotyped on an ABI 3700 XL Capillary DNA Sequencer as previously described [47,49] and SSR allele lengths were ascertained using GeneMapper (Applied Biosystems, Foster City, CA). Heterozygosities (*H*) of individual EST-SSR markers were estimated as described by Ott [50]. Genetic distances (*G*) were estimated using the proportion of shared alleles estimator in Microsat, where *G *= (1 - *p*) and *p *is the proportion of shared alleles . Neighbor-joining (NJ) trees were constructed using the NEIGHBOR program in PHYLIP  and were drawn with TreeView .

### DNA Sequence Data

Single-pass Sanger cDNA sequences (ESTs) for 6,530 *I. brevicaulis *and *I. fulva *clones have been deposited in GenBank (Acc. No. EX949962–EX956238 and FD387191–FD387443).

## Authors' contributions

ST developed the cDNA libraries, produced the ESTs, developed and screened the DNA markers, performed molecular and statistical genetic analyses, assisted with the development of the EST database and drafting the manuscript. RAO assisted with molecular analyses. MMCP, LHP, VEJ, and CAT designed and developed the EST database and performed bioinformatic analyses. MLA and SJK designed and coordinated the study and assisted with statistical analyses and drafting the manuscript.

## Supplementary Material

Additional file 1 **Perfect simple sequence repeats (1,447) identified in 1,162 unigenes in the *Iris *EST database.** Click here for file

Additional file 2 **SSR motifs and repeat counts, unigene identifiers, primer sequences, and allele lengths for 526 *Iris *EST-SSR markers.** Click here for file

Additional file 3 **Louisiana, yellow-flag, tall-bearded, and Siberian Iris ecotypes and cultivars screened for SSR allele length polymorphisms.** Click here for file

Additional file 4 **Allele length database for 40 EST-SSR markers among 39 ecotypes or cultivars of *I. brevicaulis *(IB), *I. fulva *(IF), *I. nelsonii *(IN), *I. hexagona *(IH), *I. pseudacorus *(IP), *I. germanica *(IG), and *I. sibirica *(IS).** Click here for file

Additional file 5 **Dendrogram constructed from genetic distances estimated from genotypes of 40 EST-SSR markers among seven *I. brevicaulis *(IB), six *I. fulva *(IF), six *I. hexagona *(IH), and seven *I. nelsonii *(IN) ecotypes.** Click here for file
