# Supplementary figures and images for: EST and EST-SSR marker resources for Iris
Source: BMC Plant Biol. 2009 Jun 10;9:72. doi: 10.1186/1471-2229-9-72 (PMC2703627; doi:10.1186/1471-2229-9-72)

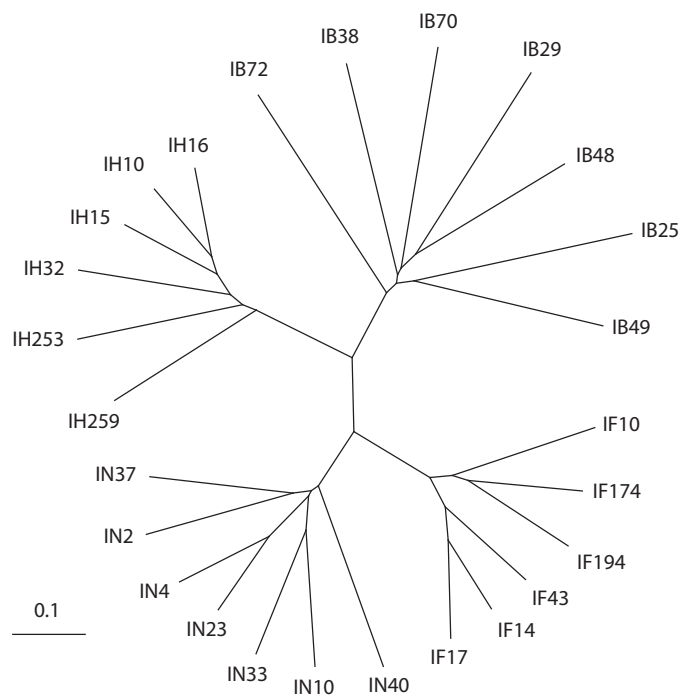

Supplement: Additional file 5 — Dendrogram constructed from genetic distances estimated from genotypes of 40 EST-SSR markers among seven I. brevicaulis (IB), six I. fulva (IF), six I. hexagona (IH), and seven I. nelsonii (IN) ecotypes. [file 1471-2229-9-72-S5.pdf]
